# (*E*)-2-[2-(4-Chloro­benzyl­idene)hydrazin­yl]-4-[3-(morpholin-4-ium-4-yl)propyl­amino]­quinazolin-1-ium bis­(perchlorate)

**DOI:** 10.1107/S1600536812021678

**Published:** 2012-05-19

**Authors:** Nan Jiang, Jian Zuo, Wenmin Zhu, Xinjie Zhao, Xin Zhai

**Affiliations:** a Key Laboratory of Original New Drug Design and Discovery of the Ministry of Education, Shenyang Pharmaceutical University, Shenyang, Liaoning 110016, People’s Republic of China; b Key Laboratory of Marine Chemistry Theory and Technology, Ministry of Education, College of Chemistry and Chemical Engineering, Ocean University of China, Qingdao, Shandong 266100, People’s Republic of China

## Abstract

In the title compound, C_22_H_27_ClN_6_O_2_^2+^·2ClO_4_^−^, the mol­ecule adopts an *E* conformation about the C=N double bond. The quinazoline ring is approximately planar, with an r.m.s. deviation of 0.0432 Å, and forms a dihedral angle of 5.77 (4)° with the chloro­phenyl ring. The crystal packing features N—H⋯O hydrogen bonds.

## Related literature

For anti­tumor background to a similar compound, see: Abouzid & Shouman (2008 ▶); Zhang *et al.* (2008 ▶); An *et al.* (2010 ▶); Horiuchi *et al.* (2009 ▶).
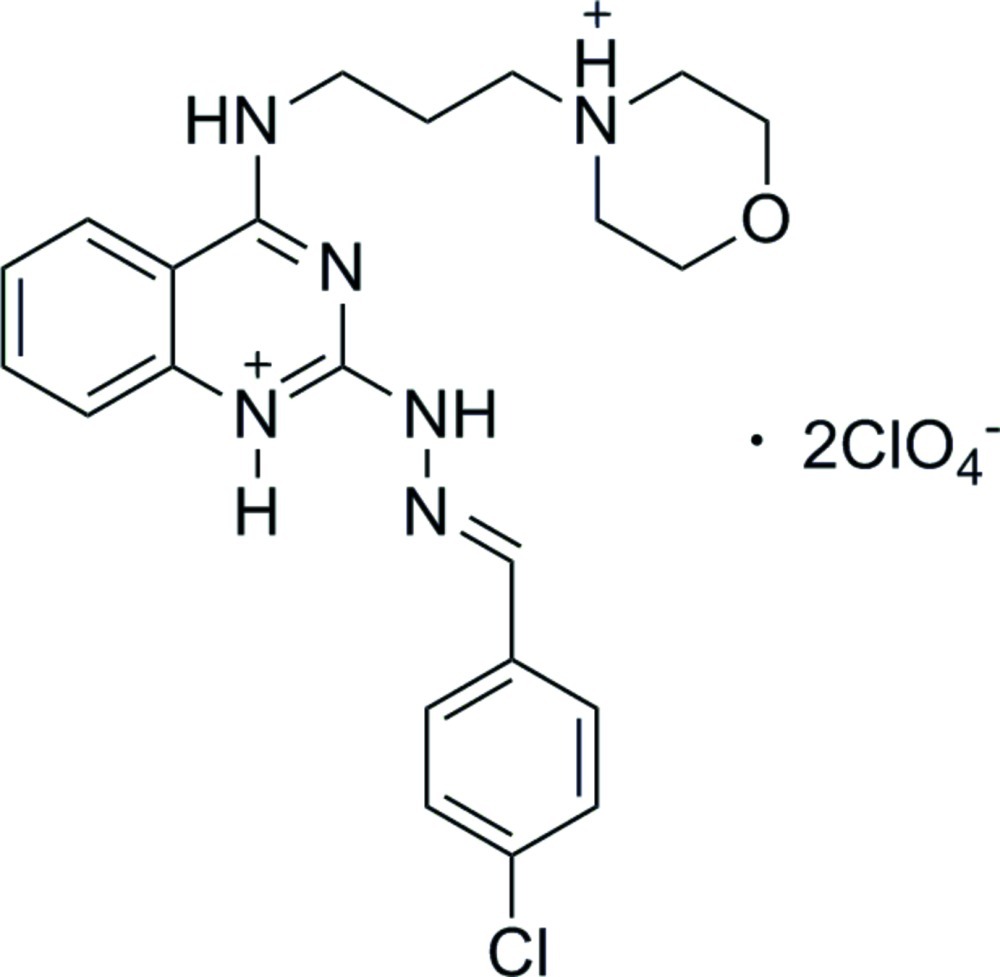


## Experimental

### 

#### Crystal data

C_22_H_27_ClN_6_O^2+^·2ClO_4_^−^
*M* *_r_* = 625.85
Monoclinic, 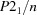

*a* = 16.1835 (7) Å
*b* = 11.6719 (4) Å
*c* = 16.7332 (6) Å
β = 112.127 (3)°
*V* = 2927.98 (19) Å^3^
*Z* = 4
Mo *K*α radiation
μ = 0.37 mm^−1^
*T* = 293 K
0.28 × 0.26 × 0.23 mm

#### Data collection

Bruker SMART CCD area-detector diffractometer
Absorption correction: multi-scan (*SADABS*; Sheldrick, 1996 ▶) *T*_min_ = 0.903, *T*_max_ = 0.920
16024 measured reflections
5127 independent reflections
2840 reflections with *I* > 2σ(*I*)
*R*_int_ = 0.083

#### Refinement

*R*[*F* ^2^ > 2σ(*F* ^2^)] = 0.070
*wR*(*F* ^2^) = 0.215
*S* = 1.02
5127 reflections
361 parameters
6 restraints
H-atom parameters constrained
Δρ_max_ = 1.13 e Å^−3^
Δρ_min_ = −0.49 e Å^−3^

### 

Data collection: *SMART* (Bruker, 1997 ▶); cell refinement: *SAINT* (Bruker, 1997 ▶); data reduction: *SAINT*; program(s) used to solve structure: *SHELXS97* (Sheldrick, 2008 ▶); program(s) used to refine structure: *SHELXL97* (Sheldrick, 2008 ▶); molecular graphics: *SHELXTL* (Sheldrick, 2008 ▶); software used to prepare material for publication: *SHELXTL*.

## Supplementary Material

Crystal structure: contains datablock(s) I, global. DOI: 10.1107/S1600536812021678/vm2162sup1.cif

Structure factors: contains datablock(s) I. DOI: 10.1107/S1600536812021678/vm2162Isup2.hkl

Supplementary material file. DOI: 10.1107/S1600536812021678/vm2162Isup3.cml

Additional supplementary materials:  crystallographic information; 3D view; checkCIF report

## Figures and Tables

**Table 1:** Hydrogen-bond geometry (Å, °)

*D*—H⋯*A*	*D*—H	H⋯*A*	*D*⋯*A*	*D*—H⋯*A*
N1—H1*C*⋯O3	0.91	1.82	2.717 (6)	168
N2—H2*C*⋯O6^i^	0.86	2.04	2.888 (5)	167
N4—H4*C*⋯O7^ii^	0.86	2.05	2.859 (5)	156
N5—H5*C*⋯O9^iii^	0.86	2.11	2.915 (5)	157

Symmetry codes: (i) 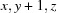; (ii) 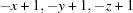; (iii) 
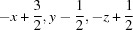
.

**Fig. 1.**
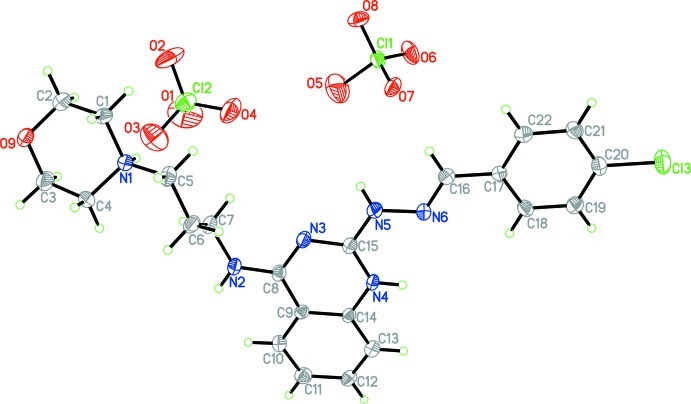
The structure of the title compound, showing 30% probability displacement ellipsoids and the atom-numbering scheme.

**Fig. 2.**
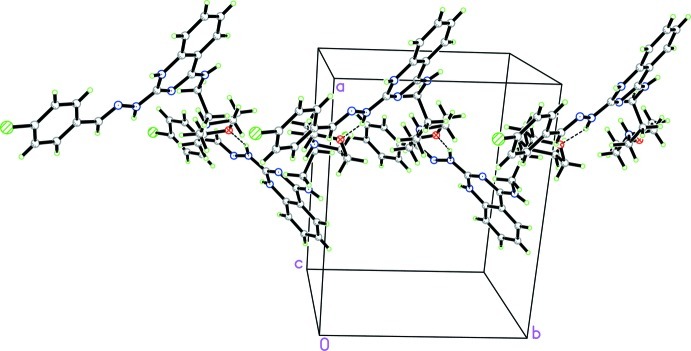
A view of the crystal packing showing a chain of molecules along the b axis linked via N—H···O contacts.

## supplementary crystallographic information

### Comment

Antitumor agents based on 4-aminoquinazoline and 4-aminoquinoline skeletons have aroused increasing attention in the last few years, such as Gefitinib and its derivatives (Abouzid *et al.*, 2008). Recently, the traditional immunostimulatory agents CQ and its derivatives, which possess the 4-aminoquinoline skeleton either, were reported for excellent antitumor potency (Zhang *et al.*, 2008; An *et al.*, 2010). In addition, hydrazone fragments are important pharmacophores which occur frequently in the design of antitumor drugs (Horiuchi *et al.*, 2009). In our ongoing research on antitumor agents with 4-aminoquinazoline and 4-aminoquinoline skeletons, the target compound, (*E*)-2-(2-(4-chlorobenzylidene)hydrazinyl)-*N*- (3-morpholinopropyl)quinazolin-4-amine diperchlorate, was designed and synthesized.

The crystal structure of the title compound is given in Fig. 1. The organic molecule adopts an E configuration about the C=N double bond. The quinazoline ring is approximately planar, with the r.m.s. deviation of 0.0432 Å. The dihedral angle between the quinazoline ring and the chlorophenyl ring is 5.77 (4) °. The molecular structure is stabilized by N1—H1C···O3, N2—H2C···O6, N4—H4C···O7 hydrogen bonds with perchlorate units (Table 1). In the crystal, adjacent molecules are stabilized by intermolecular N5—H5C···O9 hydrogen bonds linking the molecules into chains along the *b* axis (Table 1, Fig. 2).

### Experimental

Taking 2-aminobenzoic acid and urea as the starting materials, (*E*)-2-(2-(4-chlorobenzylidene)hydrazinyl)-*N*- (3-morpholinopropyl)quinazolin-4-amine was prepared according to the literature methods (Abouzid *et al.*, 2008; Horiuchi *et al.*, 2009), which was purified by silica gel column chromatography (CH_2_Cl_2_/Methanol 15:1). 70% Perchloric acid (24 mmol, 1.96 ml) was added to a solution of (*E*)-2-(2-(4-chlorobenzylidene)hydrazinyl)-*N*- (3-morpholinopropyl)quinazolin-4-amine (20 mmol, 8.5 g) in acetone (50 ml) at room temperature, then the reaction mixture was stirred at 313 K for 3 h. After cooling to ambient temperature, the mixture was filtered and washed with acetone. The resulting solid was dissolved in methanol to yield the target compound as colorless single crystals over a period of 10 days.

### Refinement

All H-atoms were positioned geometrically and refined using a riding model, with C—H = 0.93–0.97 Å, N—H = 0.86–0.91 Å, and *U*_iso_(H) =1.2*U*_eq_(C, N).

### Crystal data

**Table:**

C_22_H_27_ClN_6_O^2+^·2ClO_4_^−^	*F*(000) = 1296
*M**_r_* = 625.85	*D*_x_ = 1.420 Mg m^−^^3^
Monoclinic, *P*2_1_/*n*	Mo *K*α radiation, λ = 0.71073 Å
Hall symbol: -P 2yn	Cell parameters from 1056 reflections
*a* = 16.1835 (7) Å	θ = 3.0–19.4°
*b* = 11.6719 (4) Å	µ = 0.37 mm^−^^1^
*c* = 16.7332 (6) Å	*T* = 293 K
β = 112.127 (3)°	Block, colorless
*V* = 2927.98 (19) Å^3^	0.28 × 0.26 × 0.23 mm
*Z* = 4	

### Data collection

**Table:**

Bruker SMART CCD area-detector diffractometer	5127 independent reflections
Radiation source: fine-focus sealed tube	2840 reflections with *I* > 2σ(*I*)
Graphite monochromator	*R*_int_ = 0.083
phi and ω scans	θ_max_ = 25.0°, θ_min_ = 2.2°
Absorption correction: multi-scan (*SADABS*; Sheldrick, 1996)	*h* = −17→19
*T*_min_ = 0.903, *T*_max_ = 0.920	*k* = −13→13
16024 measured reflections	*l* = −19→19

### Refinement

**Table:**

Refinement on *F*^2^	Primary atom site location: structure-invariant direct methods
Least-squares matrix: full	Secondary atom site location: difference Fourier map
*R*[*F*^2^ > 2σ(*F*^2^)] = 0.070	Hydrogen site location: inferred from neighbouring sites
*wR*(*F*^2^) = 0.215	H-atom parameters constrained
*S* = 1.02	*w* = 1/[σ^2^(*F*_o_^2^) + (0.1136*P*)^2^] where *P* = (*F*_o_^2^ + 2*F*_c_^2^)/3
5127 reflections	(Δ/σ)_max_ < 0.001
361 parameters	Δρ_max_ = 1.13 e Å^−^^3^
6 restraints	Δρ_min_ = −0.49 e Å^−^^3^

### Special details

**Table:**

Geometry. All e.s.d.'s (except the e.s.d. in the dihedral angle between two l.s. planes) are estimated using the full covariance matrix. The cell e.s.d.'s are taken into account individually in the estimation of e.s.d.'s in distances, angles and torsion angles; correlations between e.s.d.'s in cell parameters are only used when they are defined by crystal symmetry. An approximate (isotropic) treatment of cell e.s.d.'s is used for estimating e.s.d.'s involving l.s. planes.
Refinement. Refinement of *F*^2^ against ALL reflections. The weighted *R*-factor *wR* and goodness of fit *S* are based on *F*^2^, conventional *R*-factors *R* are based on *F*, with *F* set to zero for negative *F*^2^. The threshold expression of *F*^2^ > σ(*F*^2^) is used only for calculating *R*-factors(gt) *etc*. and is not relevant to the choice of reflections for refinement. *R*-factors based on *F*^2^ are statistically about twice as large as those based on *F*, and *R*- factors based on ALL data will be even larger.

### Fractional atomic coordinates and isotropic or equivalent isotropic displacement parameters (Å^2^)

**Table:**

	*x*	*y*	*z*	*U*_iso_*/*U*_eq_	
Cl1	0.48875 (8)	0.24389 (10)	0.24289 (8)	0.0469 (4)	
Cl2	0.58604 (10)	0.68187 (13)	0.08849 (9)	0.0604 (4)	
Cl3	0.64366 (10)	0.15934 (13)	0.86537 (9)	0.0617 (5)	
O1	0.4762 (4)	0.6658 (6)	0.0106 (5)	0.149 (3)	
O2	0.6449 (3)	0.6308 (5)	0.0545 (3)	0.0987 (17)	
O3	0.5925 (4)	0.8078 (4)	0.0946 (3)	0.1027 (17)	
O4	0.5874 (3)	0.6329 (5)	0.1671 (3)	0.0980 (17)	
O5	0.4191 (4)	0.3417 (5)	0.1669 (4)	0.126 (2)	
O6	0.4327 (3)	0.1461 (3)	0.2421 (3)	0.0737 (13)	
O7	0.5211 (2)	0.3043 (3)	0.3255 (2)	0.0542 (10)	
O8	0.5596 (2)	0.2153 (3)	0.2142 (3)	0.0590 (10)	
C1	0.8016 (4)	0.9059 (5)	0.1749 (4)	0.0554 (15)	
H1A	0.8556	0.9453	0.2108	0.066*	
H1B	0.8110	0.8243	0.1855	0.066*	
C2	0.7831 (5)	0.9304 (5)	0.0821 (4)	0.0679 (18)	
H2A	0.8333	0.9056	0.0682	0.082*	
H2B	0.7309	0.8878	0.0461	0.082*	
O9	0.7683 (3)	1.0492 (3)	0.0646 (3)	0.0625 (11)	
C3	0.6953 (4)	1.0882 (5)	0.0842 (4)	0.0642 (17)	
H4A	0.6415	1.0490	0.0476	0.077*	
H4B	0.6869	1.1696	0.0720	0.077*	
C4	0.7095 (4)	1.0676 (4)	0.1775 (3)	0.0478 (13)	
H5A	0.7603	1.1118	0.2144	0.057*	
H5B	0.6574	1.0925	0.1880	0.057*	
C5	0.7460 (3)	0.9167 (4)	0.2914 (3)	0.0446 (12)	
H6A	0.8030	0.9508	0.3259	0.054*	
H6B	0.7522	0.8343	0.2988	0.054*	
C6	0.6766 (3)	0.9576 (4)	0.3258 (3)	0.0423 (12)	
H7A	0.6719	1.0403	0.3204	0.051*	
H7B	0.6970	0.9390	0.3867	0.051*	
C7	0.5840 (3)	0.9055 (4)	0.2798 (3)	0.0420 (12)	
H8A	0.5603	0.9310	0.2202	0.050*	
H8B	0.5892	0.8227	0.2798	0.050*	
C8	0.5128 (3)	0.8816 (4)	0.3846 (3)	0.0336 (11)	
C9	0.4407 (3)	0.9074 (4)	0.4136 (3)	0.0301 (10)	
C10	0.3748 (3)	0.9904 (4)	0.3750 (3)	0.0399 (12)	
H11A	0.3781	1.0362	0.3308	0.048*	
C11	0.3050 (3)	1.0038 (4)	0.4028 (3)	0.0444 (12)	
H12A	0.2609	1.0579	0.3766	0.053*	
C12	0.3006 (3)	0.9370 (4)	0.4695 (3)	0.0393 (12)	
H13A	0.2534	0.9465	0.4878	0.047*	
C13	0.3654 (3)	0.8568 (4)	0.5088 (3)	0.0399 (12)	
H14A	0.3626	0.8130	0.5541	0.048*	
C14	0.4347 (3)	0.8417 (4)	0.4807 (3)	0.0303 (10)	
C15	0.5601 (3)	0.7361 (4)	0.4838 (3)	0.0334 (11)	
C16	0.6585 (3)	0.4948 (4)	0.6029 (3)	0.0376 (11)	
H17A	0.7037	0.4866	0.5816	0.045*	
C17	0.6528 (3)	0.4124 (4)	0.6663 (3)	0.0328 (10)	
C18	0.5837 (3)	0.4168 (4)	0.6972 (3)	0.0396 (12)	
H19A	0.5393	0.4720	0.6762	0.047*	
C19	0.5810 (3)	0.3391 (4)	0.7588 (3)	0.0411 (12)	
H20A	0.5358	0.3427	0.7804	0.049*	
C20	0.6469 (3)	0.2561 (4)	0.7877 (3)	0.0394 (12)	
C21	0.7143 (3)	0.2505 (4)	0.7574 (3)	0.0416 (12)	
H22A	0.7582	0.1946	0.7777	0.050*	
C22	0.7164 (3)	0.3291 (4)	0.6964 (3)	0.0413 (12)	
H23A	0.7620	0.3252	0.6754	0.050*	
N1	0.7255 (3)	0.9444 (3)	0.1983 (2)	0.0391 (10)	
H1C	0.6759	0.9048	0.1656	0.047*	
N2	0.5224 (3)	0.9373 (3)	0.3206 (2)	0.0395 (10)	
H2C	0.4898	0.9970	0.3013	0.047*	
N3	0.5702 (2)	0.7942 (3)	0.4196 (2)	0.0365 (9)	
N4	0.4993 (2)	0.7581 (3)	0.5177 (2)	0.0345 (9)	
H4C	0.5000	0.7208	0.5623	0.041*	
N5	0.6164 (3)	0.6481 (3)	0.5155 (3)	0.0417 (10)	
H5C	0.6596	0.6362	0.4984	0.050*	
N6	0.6038 (3)	0.5771 (3)	0.5758 (2)	0.0380 (9)	

### Atomic displacement parameters (Å^2^)

**Table:**

	*U*^11^	*U*^22^	*U*^33^	*U*^12^	*U*^13^	*U*^23^
Cl1	0.0417 (7)	0.0481 (8)	0.0541 (8)	0.0074 (6)	0.0216 (6)	0.0159 (6)
Cl2	0.0563 (9)	0.0753 (11)	0.0496 (9)	0.0027 (7)	0.0201 (7)	−0.0064 (7)
Cl3	0.0664 (10)	0.0636 (9)	0.0556 (9)	0.0047 (7)	0.0238 (7)	0.0238 (7)
O1	0.099 (5)	0.156 (6)	0.161 (6)	0.003 (4)	0.013 (4)	−0.023 (5)
O2	0.073 (3)	0.165 (5)	0.072 (3)	0.045 (3)	0.043 (3)	0.001 (3)
O3	0.111 (4)	0.071 (3)	0.107 (4)	−0.028 (3)	0.020 (3)	−0.027 (3)
O4	0.089 (3)	0.149 (5)	0.074 (3)	0.048 (3)	0.051 (3)	0.051 (3)
O5	0.126 (5)	0.122 (5)	0.111 (4)	0.023 (4)	0.025 (4)	0.041 (4)
O6	0.055 (2)	0.056 (2)	0.107 (3)	−0.0075 (19)	0.027 (2)	0.030 (2)
O7	0.059 (2)	0.075 (3)	0.035 (2)	0.0039 (19)	0.0237 (18)	0.0051 (18)
O8	0.048 (2)	0.063 (2)	0.078 (3)	0.0027 (18)	0.038 (2)	−0.011 (2)
C1	0.064 (4)	0.054 (3)	0.066 (4)	0.027 (3)	0.045 (3)	0.019 (3)
C2	0.094 (5)	0.058 (4)	0.077 (4)	0.022 (3)	0.062 (4)	0.017 (3)
O9	0.078 (3)	0.059 (2)	0.075 (3)	0.023 (2)	0.057 (2)	0.026 (2)
C3	0.074 (4)	0.067 (4)	0.068 (4)	0.028 (3)	0.045 (3)	0.028 (3)
C4	0.060 (3)	0.034 (3)	0.060 (3)	0.011 (2)	0.034 (3)	0.011 (2)
C5	0.043 (3)	0.054 (3)	0.038 (3)	0.001 (2)	0.017 (2)	0.010 (2)
C6	0.047 (3)	0.047 (3)	0.041 (3)	−0.006 (2)	0.026 (2)	−0.003 (2)
C7	0.047 (3)	0.049 (3)	0.037 (3)	−0.004 (2)	0.024 (2)	0.003 (2)
C8	0.037 (3)	0.035 (3)	0.031 (3)	−0.008 (2)	0.015 (2)	−0.002 (2)
C9	0.031 (2)	0.032 (2)	0.028 (2)	−0.0028 (19)	0.012 (2)	−0.0032 (19)
C10	0.045 (3)	0.039 (3)	0.038 (3)	0.001 (2)	0.017 (2)	0.006 (2)
C11	0.039 (3)	0.049 (3)	0.046 (3)	0.008 (2)	0.016 (2)	0.002 (2)
C12	0.036 (3)	0.049 (3)	0.037 (3)	0.001 (2)	0.018 (2)	0.000 (2)
C13	0.041 (3)	0.044 (3)	0.036 (3)	−0.002 (2)	0.017 (2)	0.001 (2)
C14	0.034 (2)	0.032 (2)	0.026 (2)	−0.004 (2)	0.013 (2)	−0.0028 (19)
C15	0.035 (3)	0.036 (3)	0.030 (3)	−0.002 (2)	0.014 (2)	0.000 (2)
C16	0.040 (3)	0.038 (3)	0.038 (3)	0.000 (2)	0.018 (2)	−0.001 (2)
C17	0.037 (3)	0.031 (2)	0.032 (2)	0.003 (2)	0.016 (2)	−0.0003 (19)
C18	0.039 (3)	0.036 (3)	0.044 (3)	0.008 (2)	0.016 (2)	0.009 (2)
C19	0.040 (3)	0.045 (3)	0.042 (3)	−0.001 (2)	0.020 (2)	0.000 (2)
C20	0.046 (3)	0.036 (3)	0.034 (3)	−0.004 (2)	0.012 (2)	0.004 (2)
C21	0.043 (3)	0.041 (3)	0.038 (3)	0.009 (2)	0.012 (2)	0.005 (2)
C22	0.040 (3)	0.042 (3)	0.045 (3)	0.006 (2)	0.020 (2)	0.000 (2)
N1	0.045 (2)	0.036 (2)	0.043 (2)	0.0045 (18)	0.024 (2)	0.0054 (18)
N2	0.045 (2)	0.040 (2)	0.042 (2)	0.0005 (18)	0.025 (2)	0.0079 (18)
N3	0.038 (2)	0.043 (2)	0.033 (2)	0.0021 (19)	0.0182 (18)	0.0058 (18)
N4	0.042 (2)	0.038 (2)	0.030 (2)	0.0042 (18)	0.0201 (18)	0.0085 (17)
N5	0.045 (2)	0.043 (2)	0.045 (2)	0.0109 (19)	0.026 (2)	0.0099 (19)
N6	0.048 (2)	0.034 (2)	0.037 (2)	0.0015 (19)	0.021 (2)	0.0052 (17)

### Geometric parameters (Å, º)

**Table:**

Cl1—O8	1.440 (3)	C8—N3	1.355 (6)
Cl1—O6	1.455 (4)	C8—C9	1.455 (6)
Cl1—O7	1.462 (4)	C9—C14	1.393 (6)
Cl1—O5	1.764 (5)	C9—C10	1.404 (6)
Cl2—O2	1.412 (4)	C10—C11	1.384 (6)
Cl2—O4	1.427 (4)	C10—H11A	0.9300
Cl2—O3	1.474 (5)	C11—C12	1.385 (7)
Cl2—O1	1.774 (7)	C11—H12A	0.9300
Cl3—C20	1.737 (5)	C12—C13	1.374 (6)
C1—C2	1.493 (7)	C12—H13A	0.9300
C1—N1	1.496 (6)	C13—C14	1.382 (6)
C1—H1A	0.9700	C13—H14A	0.9300
C1—H1B	0.9700	C14—N4	1.393 (6)
C2—O9	1.418 (6)	C15—N3	1.331 (5)
C2—H2A	0.9700	C15—N4	1.332 (5)
C2—H2B	0.9700	C15—N5	1.343 (6)
O9—C3	1.415 (6)	C16—N6	1.269 (6)
C3—C4	1.509 (7)	C16—C17	1.461 (6)
C3—H4A	0.9700	C16—H17A	0.9300
C3—H4B	0.9700	C17—C22	1.366 (6)
C4—N1	1.478 (6)	C17—C18	1.398 (6)
C4—H5A	0.9700	C18—C19	1.387 (6)
C4—H5B	0.9700	C18—H19A	0.9300
C5—N1	1.500 (6)	C19—C20	1.387 (7)
C5—C6	1.518 (6)	C19—H20A	0.9300
C5—H6A	0.9700	C20—C21	1.366 (7)
C5—H6B	0.9700	C21—C22	1.383 (6)
C6—C7	1.530 (7)	C21—H22A	0.9300
C6—H7A	0.9700	C22—H23A	0.9300
C6—H7B	0.9700	N1—H1C	0.9100
C7—N2	1.453 (6)	N2—H2C	0.8600
C7—H8A	0.9700	N4—H4C	0.8600
C7—H8B	0.9700	N5—N6	1.379 (5)
C8—N2	1.311 (5)	N5—H5C	0.8600
			
O8—Cl1—O6	112.8 (2)	C14—C9—C8	117.6 (4)
O8—Cl1—O7	112.4 (2)	C10—C9—C8	123.7 (4)
O6—Cl1—O7	112.7 (2)	C11—C10—C9	119.8 (4)
O8—Cl1—O5	106.4 (3)	C11—C10—H11A	120.1
O6—Cl1—O5	105.9 (3)	C9—C10—H11A	120.1
O7—Cl1—O5	105.9 (3)	C10—C11—C12	120.3 (5)
O2—Cl2—O4	115.1 (3)	C10—C11—H12A	119.9
O2—Cl2—O3	114.1 (3)	C12—C11—H12A	119.9
O4—Cl2—O3	111.1 (3)	C13—C12—C11	120.5 (5)
O2—Cl2—O1	107.5 (3)	C13—C12—H13A	119.7
O4—Cl2—O1	107.3 (4)	C11—C12—H13A	119.7
O3—Cl2—O1	100.4 (3)	C12—C13—C14	119.7 (4)
C2—C1—N1	110.6 (4)	C12—C13—H14A	120.2
C2—C1—H1A	109.5	C14—C13—H14A	120.2
N1—C1—H1A	109.5	C13—C14—C9	121.1 (4)
C2—C1—H1B	109.5	C13—C14—N4	120.5 (4)
N1—C1—H1B	109.5	C9—C14—N4	118.4 (4)
H1A—C1—H1B	108.1	N3—C15—N4	125.5 (4)
O9—C2—C1	110.7 (5)	N3—C15—N5	115.8 (4)
O9—C2—H2A	109.5	N4—C15—N5	118.7 (4)
C1—C2—H2A	109.5	N6—C16—C17	122.1 (4)
O9—C2—H2B	109.5	N6—C16—H17A	119.0
C1—C2—H2B	109.5	C17—C16—H17A	119.0
H2A—C2—H2B	108.1	C22—C17—C18	119.1 (4)
C3—O9—C2	111.0 (4)	C22—C17—C16	119.4 (4)
O9—C3—C4	111.9 (5)	C18—C17—C16	121.5 (4)
O9—C3—H4A	109.2	C19—C18—C17	120.3 (4)
C4—C3—H4A	109.2	C19—C18—H19A	119.8
O9—C3—H4B	109.2	C17—C18—H19A	119.8
C4—C3—H4B	109.2	C20—C19—C18	118.7 (4)
H4A—C3—H4B	107.9	C20—C19—H20A	120.6
N1—C4—C3	110.2 (4)	C18—C19—H20A	120.6
N1—C4—H5A	109.6	C21—C20—C19	121.4 (4)
C3—C4—H5A	109.6	C21—C20—Cl3	120.0 (4)
N1—C4—H5B	109.6	C19—C20—Cl3	118.6 (4)
C3—C4—H5B	109.6	C20—C21—C22	119.2 (4)
H5A—C4—H5B	108.1	C20—C21—H22A	120.4
N1—C5—C6	114.8 (4)	C22—C21—H22A	120.4
N1—C5—H6A	108.6	C17—C22—C21	121.3 (5)
C6—C5—H6A	108.6	C17—C22—H23A	119.3
N1—C5—H6B	108.6	C21—C22—H23A	119.3
C6—C5—H6B	108.6	C4—N1—C1	108.6 (4)
H6A—C5—H6B	107.5	C4—N1—C5	113.9 (4)
C5—C6—C7	114.1 (4)	C1—N1—C5	109.0 (4)
C5—C6—H7A	108.7	C4—N1—H1C	108.4
C7—C6—H7A	108.7	C1—N1—H1C	108.4
C5—C6—H7B	108.7	C5—N1—H1C	108.4
C7—C6—H7B	108.7	C8—N2—C7	124.8 (4)
H7A—C6—H7B	107.6	C8—N2—H2C	117.6
N2—C7—C6	112.2 (4)	C7—N2—H2C	117.6
N2—C7—H8A	109.2	C15—N3—C8	117.4 (4)
C6—C7—H8A	109.2	C15—N4—C14	119.8 (4)
N2—C7—H8B	109.2	C15—N4—H4C	120.1
C6—C7—H8B	109.2	C14—N4—H4C	120.1
H8A—C7—H8B	107.9	C15—N5—N6	118.9 (4)
N2—C8—N3	117.0 (4)	C15—N5—H5C	120.6
N2—C8—C9	121.8 (4)	N6—N5—H5C	120.6
N3—C8—C9	121.1 (4)	C16—N6—N5	116.1 (4)
C14—C9—C10	118.7 (4)		
			
N1—C1—C2—O9	58.6 (7)	C18—C19—C20—Cl3	179.7 (4)
C1—C2—O9—C3	−59.2 (7)	C19—C20—C21—C22	−0.4 (7)
C2—O9—C3—C4	58.6 (7)	Cl3—C20—C21—C22	−179.2 (4)
O9—C3—C4—N1	−57.0 (6)	C18—C17—C22—C21	−0.9 (7)
N1—C5—C6—C7	61.1 (6)	C16—C17—C22—C21	179.0 (4)
C5—C6—C7—N2	173.8 (4)	C20—C21—C22—C17	0.4 (7)
N2—C8—C9—C14	178.4 (4)	C3—C4—N1—C1	54.8 (6)
N3—C8—C9—C14	2.2 (6)	C3—C4—N1—C5	176.4 (4)
N2—C8—C9—C10	1.8 (7)	C2—C1—N1—C4	−56.3 (6)
N3—C8—C9—C10	−174.4 (4)	C2—C1—N1—C5	179.1 (5)
C14—C9—C10—C11	−1.2 (7)	C6—C5—N1—C4	55.1 (6)
C8—C9—C10—C11	175.4 (4)	C6—C5—N1—C1	176.5 (4)
C9—C10—C11—C12	1.0 (7)	N3—C8—N2—C7	6.0 (7)
C10—C11—C12—C13	0.1 (7)	C9—C8—N2—C7	−170.4 (4)
C11—C12—C13—C14	−1.0 (7)	C6—C7—N2—C8	−87.4 (6)
C12—C13—C14—C9	0.8 (7)	N4—C15—N3—C8	−1.4 (7)
C12—C13—C14—N4	−177.7 (4)	N5—C15—N3—C8	178.2 (4)
C10—C9—C14—C13	0.3 (6)	N2—C8—N3—C15	−179.0 (4)
C8—C9—C14—C13	−176.5 (4)	C9—C8—N3—C15	−2.6 (6)
C10—C9—C14—N4	178.8 (4)	N3—C15—N4—C14	5.7 (7)
C8—C9—C14—N4	2.0 (6)	N5—C15—N4—C14	−173.9 (4)
N6—C16—C17—C22	−176.6 (4)	C13—C14—N4—C15	172.7 (4)
N6—C16—C17—C18	3.3 (7)	C9—C14—N4—C15	−5.8 (6)
C22—C17—C18—C19	1.4 (7)	N3—C15—N5—N6	−173.4 (4)
C16—C17—C18—C19	−178.5 (4)	N4—C15—N5—N6	6.3 (6)
C17—C18—C19—C20	−1.3 (7)	C17—C16—N6—N5	−179.6 (4)
C18—C19—C20—C21	0.8 (7)	C15—N5—N6—C16	179.4 (4)

### Hydrogen-bond geometry (Å, º)

**Table:**

*D*—H···*A*	*D*—H	H···*A*	*D*···*A*	*D*—H···*A*
N1—H1*C*···O3	0.91	1.82	2.717 (6)	168
N2—H2*C*···O6^i^	0.86	2.04	2.888 (5)	167
N4—H4*C*···O7^ii^	0.86	2.05	2.859 (5)	156
N5—H5*C*···O9^iii^	0.86	2.11	2.915 (5)	157

Symmetry codes: (i) *x*, *y*+1, *z*; (ii) −*x*+1, −*y*+1, −*z*+1; (iii) −*x*+3/2, *y*−1/2, −*z*+1/2.
